# Suubi+Adherence-Round 2: A study protocol to examine the longitudinal HIV treatment adherence among youth living with HIV transitioning into young adulthood in Southern Uganda

**DOI:** 10.1186/s12889-021-10202-3

**Published:** 2021-01-21

**Authors:** Fred M. Ssewamala, Ozge Sensoy Bahar, Proscovia Nabunya, April D. Thames, Torsten B. Neilands, Christopher Damulira, Barbara Mukasa, Rachel Brathwaite, Claude Mellins, John Santelli, Derek Brown, Shenyang Guo, Phionah Namatovu, Joshua Kiyingi, Flavia Namuwonge, Mary M. McKay

**Affiliations:** 1 Brown School, Washington University in St. Louis, 1 Brookings Drive, St. Louis, MO 63130 USA; 2 Department of Psychology, University of Southern California, 3620 S. McClintock Avenue Rm 520, Los Angeles, CA 90089 USA; 3 School of Medicine, University of California San Francisco, 550 16th Street, San Francisco, CA 94158 USA; 4 Mildmay Uganda, 12 Km Entebbe Road, Naziba Hill, Lweza, Kampala, Uganda; 5 HIV Center for Clinical & Behavioral Studies, New York State Psychiatric Institute and Columbia University, 1051 Riverside Dr, New York, NY 10032 USA; 6 Department of Population and Family Health, Columbia University Mailman School of Public Health, 60 Haven Ave B-4 Suite 432, New York, NY 10032 USA; 7 International Center for Child Health and Development Field Office, Plot 23 Circular Rd, Masaka, Uganda

**Keywords:** Youth living with HIV, cohort, economic empowerment, sub-Saharan Africa, Uganda, HIV/AIDS

## Abstract

**Background:**

Youth living with HIV (YLHIV) in Sub-Saharan African (SSA) are less likely to adhere to antiretroviral therapy (ART) and other health-related regimens. As a consequence, YLHIV are not only at risk for health problems and mental health comorbidities, but are also at risk for cognitive deficits, including in areas of memory and executive functioning. The Suubi+Adherence study followed 702 adolescents (10-16 years of age) receiving bolstered standard of care and a family economic empowerment intervention comprising an incentivized youth financial savings account (YSA) augmented with financial literacy training (FLT) and peer mentorship. The study findings pointed to superior short-term viral suppression and positive adolescent health and mental health functioning among participants receiving the intervention. The original group of adolescents who received Suubi+Adherence are now transitioning into young adulthood. This paper presents a protocol for the follow-up phase titled Suubi+Adherence Round 2.

**Methods:**

The original cohort in Suubi+Adherence will be tracked for an additional five years (2020-2025). Specifically, the long term follow-up will allow to: 1) ascertain the extent to which the short term outcomes identified in the first 6 years of the intervention are maintained as the same group transitions through young adulthood; and 2) address new scientific questions regarding ART adherence; HIV care engagement; protective health behaviors; and the potential of FEE to mitigate the development of HIV-associated neurocognitive disorders in YLHIV. Additionally, the team examines the potential mechanisms through which the observed long-term outcomes happen. Moreover, the Suubi+Adherence-Round 2 adds a qualitative component and extends the cost effectiveness component.

**Discussion:**

Guided by asset and human development theories, Suubi+Adherence-R2 will build on the recently concluded Suubi+Adherence study to conduct one of the largest and longest running studies of YLHIV in SSA as they transition into young adulthood. The study will address new scientific questions regarding long-term ART adherence, HIV care engagement, protective health behaviors, and the potential of FEE to mitigate the development of HIV-associated neurocognitive disorders in YLHIV. The findings may inform efforts to improve HIV care among Uganda’s YLHIV, with potential replicability in other low-resource countries.

**Trial registration:**

ClinicalTrials.gov, ID: NCT01790373

## Introduction

Sub Saharan Africa (SSA) has the highest HIV prevalence rate in the world [1]. A recent HIV assessment in Uganda, a poor SSA country hardest hit with HIV, puts the prevalence of viral load suppression among adolescents and young adults (15 to 24 years old) to be 44.9% for females and 32.5% for males [2]. For six years (2012-2018), the *Suubi+Adherence* study team prospectively followed 702 youth living with HIV (YLHIV; aged 10 to 16 years at enrollment) across 39 clinics in Uganda testing the impact of a family economic empowerment (FEE) intervention comprising an incentivized youth financial savings account (YSA) augmented with financial literacy training (FLT) and peer mentorship [3]. Our study findings pointed to superior short-term viral suppression and positive child health and mental health functioning among the intervention arm receiving the FEE intervention compared to the control arm [4–7]. This study proposes to examine whether the results are maintained through young adulthood, an incredibly vulnerable transition period [8], particularly in areas of cognitive development, mental health, sexual risk taking behaviors and alcohol/drug misuse [9–12]. Moreover, given the stigma of HIV, YLHIV report lower social support, and increased risk for non-adherence as they become more autonomous [13, 14]. Guided by two complementary theories related to assets [15–17], and human development [17, 18] applied within a life course perspective [17], this study (*Suubi+Adherence-Round 2)* will build on the recently concluded *Suubi+Adherence* study to conduct one of the largest and longest running studies of YLHIV in SSA during a developmental period marked by profound biological and psychological maturation, and development transitional milestones (e.g., leaving home, school completion, workforce entry, romantic partnerships/marriage, parenthood) [17, 18]. The study has the following specific aims:

### Aim 1

To examine the long-term impact of the *Suubi+Adherence* intervention on: HIV viral suppression (primary outcome) and to explore in secondary analyses the long-term impact of the intervention on key HIV treatment adherence outcomes for YLHIV, including participants’ ability to access and refill prescribed medication, adhere to prescribed daily medication routines, and engage in HIV care (e.g., keeping medical appointments).

### Aim 2

To elucidate the long-term effects of the *Suubi+Adherence* intervention on potential mechanisms of change, including: a) economic stability, sexual risk-taking behavior, adherence self-efficacy; b) cognitive functioning (global cognition as well as cognitive domains of memory, and executive functioning) using cognitive tests previously validated in Uganda among YLHIV [19, 20]; c) mental health functioning (hopelessness, depression, self-efficacy); and d) young adult transitions and social support.

### Aim 3

To qualitatively examine– prospectively and retrospectively–: a) multi-level factors affecting participants’ maintenance of intervention benefits since *Suubi+Adherence* intervention initiation (prospectively); and b) participants’ experiences with the intervention (retrospectively), including multi-level factors that may have influenced their engagement with the program, as well as their decision-making in regard to ART adherence.

### Aim 4

To examine the long-term cost-effectiveness of the *Suubi+Adherence* intervention.

## Background

Sub-Saharan African (SSA) has the highest HIV prevalence rate in the world, including large numbers of children born with HIV prior to the widespread roll out of antiretroviral therapy (ART) [21]. Due to treatment advances, and with increasing access to ART [22], a generation of youth living with HIV (YLHIV) in SSA hitherto not expected to reach their 5th birthday are transitioning into young adulthood. Although prevention of mother to child HIV transmission has resulted in fewer perinatal HIV infections, 3.3 million children <15 years of age are living with HIV globally; the majority are approaching young adulthood [23]. Many of these children experience compromised health, inconsistent ART adherence, elevated mental health difficulties, and risk behaviors with public health consequences (e.g. HIV transmission to others) [8–10, 24–28] Uganda, one of the countries hardest hit with HIV in SSA, reports unprecedented numbers of YLHIV. Over 180,000 children (ages 0-14) are living with HIV in Uganda [29, 30]. As ART has become more readily available (free ARV roll-out in Uganda began in 2004 [30], the consequences have gradually changed with a decrease in mortality and increased likelihood that a growing number of YLHIV will now cope with HIV as a chronic, highly stigmatized, and transmittable illness [31–33].

A recent HIV assessment in Uganda, a poor SSA country hardest hit with HIV, puts the prevalence of viral load suppression among adolescents and young adults (15 to 24 years old) to be 44.9% for females and 32.5% for males [2]. For six years (2012-2018), the *Suubi+Adherence* study team prospectively followed 702 YLHIV (aged 10 to 16 years at enrollment) randomized to two study arms across 39 clinics in Uganda: 1) control arm consisting of bolstered standard of care (BSOC) and 2) intervention arm consisting of BSOC and a FEE intervention comprising an incentivized youth financial savings account (YSA) augmented with financial literacy training (FLT) and peer mentorship (see Suubi+Adherence protocol paper [3] for more details). Our study findings pointed to superior short-term viral suppression and positive child health and mental health functioning among the intervention arm receiving the FEE intervention compared to the control arm [3–6]. The *Suubi+Adherence-Round 2* study will examine whether the results are maintained through young adulthood, an incredibly vulnerable transition period [8], particularly in areas of cognitive development, mental health, sexual risk taking behaviors and alcohol/drug misuse [9–12]. Moreover, given the stigma of HIV, YLHIV report lower social support, and increased risk for non-adherence as they become more autonomous [13, 14].

### Young Adulthood as a Vulnerable Developmental Period

Young adulthood, typically defined as ages 18-29 [9], is one of the most challenging transition periods [24] and is characterized by identity formation as well as a feeling of existing “in between” adolescence and adult-stages [24, 28]. Social and behavioral scientists discuss young adulthood in terms of five major role transitions: leaving home, completing school, entering the workforce, forming a romantic partnership, and transitioning to parenthood [17, 18]. The ordering, timing, and tempo of these transitions can have health consequences, including suboptimal adherence to long-term prescribed medications, and HIV risk behaviors. The transition to young adulthood occurs in the context of fewer social controls. It is a period when youth initiate adult roles and responsibilities [34–36] and establish patterns of positive and risky health behaviors that carry through to adulthood [37–39]. Some of the freedoms youth acquire during this stage encourage exploration and experimentation. Moreover, due to this age group’s unique adversities, increased likelihood of risk-taking behaviors and lower perceptions of social support, it is also susceptible to compromised mental health [9, 40] and cognitive functioning [41]. Specifically for youth living with HIV (YLHIV), this stage is associated with the lowest ART adherence levels [42–51] and increased substance use and health-risking behaviors [26, 52, 53]. Successfully accomplishing developmental tasks during young adulthood not only influences immediate functioning, but also lays the foundation for optimal functioning later in life [18, 54]. Young adulthood is an opportunity to promote healthy development [55, 56], to alter negative pathways from childhood and adolescence, and optimize successful transition to adulthood [34]. Yet, very little is known about YLHIV who are also managing a chronic, transmittable, and stigmatizing illness, especially in a poor country like Uganda—the focus of this application. This is an important period for interventions to promote health, treatment engagement, and successful young adult transition, yet few interventions target this age group [57–60].

### Risk Factors against Successful Transition to Adulthood among YLHIV

Since combination ART was not routinely available to children in many SSA countries until mid 2000s, many YLHIV in the region approaching young adulthood have endured years of sub-optimal treatment and are at risk for active neurotropic and neuroinflammatory HIV disease [61, 62]. Neurocognitive deficits in YLHIV affect school achievement, relationships and autonomy [63]. HIV may affect subcortical white matter and frontostriatal systems involved in regulation of emotion and behavior [64, 65], placing youth at risk for mental health problems in young adulthood [66]. For youth exposed to early severe HIV disease, psychosocial ramifications of hospitalizations, potential mortality, missed school and social opportunities, and delayed puberty are significant [31]. These early deficits and experiences – even in the context of reconstituted immune systems – may profoundly limit the ability of YLHIV to complete high school, find employment, have relationships, and function independently as they transition into young adulthood [26, 52, 53, 64, 66–68]. Furthermore, the vast majority of YLHIV in SSA are from poor families, living in impoverished communities with disruptions in caregiving due to parental illness or death. This adversity is compounded by living with a lifelong, transmittable and chronic illness. U.S. studies of YLHIV indicate higher rates of psychiatric disorders and emotional and behavioral problems than in other youth populations [31, 69] and increased substance use [70–73]. Furthermore, mental health problems often increase significantly in young adulthood. For YLHIV in low resourced communities of SSA and transitioning into young adulthood, the circumstances may be worse. Yet, we do not know much about this group because, generally, to date HIV prevention, care and support intervention efforts in SSA communities have primarily been “transported” from outside the region, mainly from the global north [2, 69, 74–76].

### High Risk for ART Non-adherence

YLHIV are highly susceptible to non-adherence to treatment, especially if they live in low-resource settings [42–51]. Recent data from SSA indicate that adherence may be one of the greatest barriers to realizing the full benefits of ART [77–81]. When people fail to adhere to their prescribed treatment, the viral loads increase and their immune systems become resistant to first-line HIV drugs, yet second-line drugs are expensive and unavailable in much of SSA. Studies have documented the strong relationship between high ART adherence and better virologic, immunologic, and clinical outcomes [26, 53, 82] with poor adherence leading to inadequate viral suppression – a serious problem for the individual (leading to clinical and immunological decline and development of drug resistant viral strains) [80, 83, 84] and for the public health (the potential for transmission of drug-resistant viral strains to others) [78, 85–88]. The prevalence of viral load suppression is distinctly lower among younger adults: 44.9% among HIV-positive females and 32.5% among HIV-positive males aged 15 to 24 years [2]. Hence, failure to address ART adherence needs of YLHIV transitioning into adulthood may lead to costly long-term consequences [14, 33, 89].

Financial instability is a barrier to ART adherence in low-resource settings [42–51, 90–94]. Specifically in SSA, individual and family-level financial instability, including lack of assets, monetary income, and material resources, deter people living with HIV/AIDS from adhering to their prescribed regimen. Furthermore, people often cease taking their prescribed HIV medications due to the inability to address a significant side effect [44, 51, 91]. An increased appetite is a side-effect of ART, and ART requires greater caloric consumption, especially when patients initiate ART. An increased appetite can have serious implications for people in SS [43, 51, 91] For impoverished families, there will be an increased burden on limited resources when families cannot meet this demand. Studies in SSA report fear of increased appetite and not having sufficient food as barriers to ART adherence [46, 47, 51]. Further, financial instability impacts ART adherence through transportation costs to health clinics. Studies from SSA, including Uganda, show that the costs of transportation to health clinics impeded individuals from attending their scheduled follow-up appointments and obtaining refills of ARVs [46, 48]. As a result, many patients have gaps in their treatment regimens and often cease taking ARVs for long periods of time. Gaps in treatment increase the risk of virologic failure and resistance to first-line HIV drugs [95], yet second-line drugs are expensive and unavailable in many resource constrained settings. In resource-constrained settings, YLHIV sacrifice healthcare, including adherence to treatment, and other basic needs (e.g., food, clothing, and school fees) [42, 46, 47, 49, 50].

### Economic Instability, Treatment Adherence, Mental Health, and Neurocognitive Functioning

Young people impacted by HIV (including YLHIV) in Uganda often live in poverty and show high rates of compromised neurocognitive development, depression, anxiety, learning problems, and sexual risk-taking [96–103]. They often experience low self-esteem and hopelessness, which can negatively influence decisions about substance use and/or sexual risk-taking, further increasing HIV vulnerability. Moreover, young people residing in low-resource communities have lower levels of secondary education attendance, higher rates of low-wage work, and more young parenting [25, 104], resulting in negative outcomes (e.g., unemployment and mental health problems) that compromise successful social transitions and ART adherence. Given the poverty and disproportionate HIV rates in much of SSA, it is critical to delineate the health and wellbeing needs of YLHIV who reside in this region. When families are poor, even if governments make ARVs free, there are costs that they have to deal with (e.g., transport, food, managing the side effects that may occur). The provision of psychosocial counseling alone –emphasized by standard of care - does not eliminate financial constraints, a risk factor for non-adherence faced by many YLHIV in SSA, especially during a developmental period marked by profound biological and psychological maturation, and development transitional milestones [17, 18]. Thus, research to understand longitudinal outcomes related to ART adherence and virologic suppression as well as the potential mechanisms of protective health behaviors among YLHIV during their social transitions in a poor region like SSA is essential.

Moreover, targeting adherence in YLHIV has the potential to mitigate the development of HIV-associated neurocognitive disorders [105–107]. ART initiation and adherence are associated with better cognitive outcomes in both youth and adult populations, including a reduction in the prevalence and severity of HIV-associated neurocognitive disorders (HAND) and HIV neuropathology. Neuropsychological deficits in executive functioning, attention, and verbal memory have been associated with poor adherence [105–107]. Therefore, early and consistent treatment with ART following HIV diagnosis could promote continued adherence [106, 107].

### Theoretical Models Guiding the Study

#### Asset theory

[15, 16] posits that assets-ownership can lead to wide scale benefits, including expectations for more resources in the future, optimistic thinking, feelings of safety and security [108], and future planning [15, 16, 109]. Asset building refers to efforts that enable people with limited economic opportunities to acquire and accumulate long-term productive assets [110]. It is increasingly viewed as a critical factor for reducing poverty, positively impacting attitudes and behaviors, and improving psychosocial functioning and stability [72, 111, 112]. Asset-theory is consistent with several behavioral and psychosocial theories (e.g., Bandura’s Social Cognitive Theory [113] and the Theory of Reasoned Action) [114–119]. Asset theory suggests many direct and indirect positive effects on individuals and families from asset ownership, and contributes to understanding how attitudes and beliefs evolve, which in turn influence intentions and behaviors [15].

#### Developmental theory

YLHIV are affected by parental HIV, orphanhood, poverty, stigma and discrimination [1, 8, 96, 97, 120]. These experiences may interfere with normal development (including cognitive development) and disrupt the healthy transition through developmental stages, resulting in disengagement from opportunities (including healthcare opportunities), school failure, risk-taking behaviors (sexual risk-taking and alcohol/drug use), poor emotional well-being, and poverty [121, 122]. Moreover, lack of family support may make developmental transitions more difficult [121, 122]. Thus, interventions aimed at supporting YLHIV should focus on increasing self-efficacy and enhanced control over one’s life [121, 122]. This is the focus of the *Suubi+Adherence* intervention. This extension, therefore, offers a time-sensitive and unique opportunity to examine the long-term effects of small direct investments on young people through an asset-oriented FEE package as they transition into young adulthood**).**

#### Progress of the *Suubi+Adherence* study (original study)

To our knowledge, the *Suubi+Adherence* study was the first to incorporate and test a savings-led FEE intervention for economic stability into the commonly used/standard adherence counseling practices in Uganda. Before *Suubi+Adherence*, we did not know the extent to which the same extremely promising Suubi FEE intervention tested in our earlier studies in Uganda, including Seed-Uganda (2004) [74, 123], Suubi-Uganda (2005-2008) [101, 103, 124] and Suubi-Maka (2008-2011) [96, 97, 102, 125–128], which together informed the *Suubi+Adherence* study (2012-2018) would perform in addressing critical health behaviors required to manage HIV as a chronic illness among YLHIV. The *Suubi+Adherence* study findings, however, advanced knowledge in regards to short-term outcomes; used rigorous objective measures, i.e. biomarkers (viral suppression) [7] and unannounced pill counts, and included a cost-effectiveness analysis.

The *Suubi+Adherence* study filled important gaps on the effect of FEE interventions on adherence to ART. More specifically, study data made significant contributions to the very limited number of evidence-based FEE interventions evaluated to address ART adherence among YLHIV in low-resource settings. In addition, study findings created space for key research and policy dialogue on the intersection between economic stability and HIV treatment [129, 130]. In *Suubi+Adherence*, adolescents’ adherence to ART was measured using viral load (VL) suppression. Viral load testing was done at baseline,12, 24, 36 and 48-months post intervention initiation. In accordance with the Abbott platform, VL was dichotomized between undetectable (< 40 copies/ml) and detectable (≥ 40 copies/ml) levels. Across the study period, we found that the proportion of virally suppressed participants in the intervention condition steadily increased. For example, we found significantly lower odds of youth in the intervention condition having a detectable VL at both 12- and 24-months [7]. Overall, the findings indicate that in the short-term, EE interventions have the potential to bolster ART-related health outcomes such as HIV viral suppression by improving ART adherence among vulnerable adolescents living in low-resource environments [7]. This is the desired outcome in ART adherence.

In another analysis [5], we assessed whether missing ART medication doses among our participants was significantly associated with individual- and community-level indicators of inequities, including economic (availability of household assets, employment and food security), social support, and structural inequities (participants’ access and proximity to community resources, including schools, and health clinics). These are important questions as they relate to social transitions YLHIV go through. Greater asset ownership (a proxy for financial stability) was associated with greater odds of self-reported adherence (OR 1.69, 95% CI: 1.00–2.85). Also, we found that distance to the nearest health clinic impacts youth’s adherence to an ARV, with youth who reported living nearer to a clinic more likely to report optimal adherence (OR 1.49, 95% CI: 0.92–2.40). Moreover, we found that youth with greater economic advantage in assets-ownership and financial savings report higher odds of adherence (OR 1.70, 95% CI: 1.07–2.70) [5]. Specific to financial savings, the mean savings for participants in the intervention arm increased from USD equivalent of $2.15 at baseline to $19.34 at 24-months, while the control arm participants’ mean savings increased from $1.78 to $4.44 over the same time period.

Applying an intent-to-treat analysis, and using multilevel logistic regressions comparing the effect of the intervention on participants in the treatment condition to participants in the control condition and using viral load suppression (< 40 copies/ml) at 24 months as the primary outcome, we calculate per-participant costs for each study condition. We then use intervention effects and per-participant costs to compute incremental cost-effectiveness ratios from a provider perspective. We find that at 24 months post intervention initiation, per-participant cost was US$109 for the intervention condition and US$27 for the control condition. While the estimated cost of achieving 10% increase in the probability of being virally suppressed was US$71 (95% CI $30, $680), the cost per virally suppressed adolescent was estimated at $923 (95% CI $474, $2982). In sum, our findings indicate that the Suubi+Adherence intervention was effective in improving ART adherence among YLHIV and the cost per virally suppressed adolescent is not prohibitive. Indeed, the findings are significant given the limited evidence regarding the cost-effectiveness of medication adherence for HIV. Moreover, in a separate incidence analysis [6], we use baseline data from adolescents with detectable VL. We found that over time, the incidence of undetectable VL among participants in the intervention condition increased significantly compared to participants in the control condition (adj. HR=1.56, CI: 1.18-2.06, p=0.01). Moreover, our analysis of the final data, located a borderline significant higher hazard for undetectable HIV VL among girls (HR=1.269, CI: 1.003 - 1.610, p=0.05) in comparison to boys while controlling for family structure and other demographics characteristics.

Overall, we find that in the short-term Suubi+Adherence produced several desired outcomes for ART adherence. The question, however, remains as for how long the reported outcomes will be sustained through young adulthood. Thus, there is a need for a longer-term follow-up period to establish how the results are impacted across the years as participants go through social transitions – a very vulnerable stage for adherence – and the associated costs and cost effectiveness.

## Methods

### Overview of the *Suubi+Adherence* Study (original study)

The goal of *the Suubi+Adherence* study was to examine the impact and cost of an innovative EE intervention to increase adherence to HIV treatment for HIV-infected youth (see Suubi+Adherence study protocol [3] for more details). The study was guided by asset-theory [15, 16] and tested a FEE intervention consisting of an incentivized youth financial savings accounts (YSA) augmented with peer mentorship, financial literacy training (FLT) and income generating activities (IGAs) for poverty-impacted youth living with HIV and their families. The intervention was intended to: 1) economically stabilize families to yield sufficient income to meet the needs of managing HIV as a chronic illness (e.g. having sufficient nutritional resources, and funds to cover costs associated with medical care and; 2) provide support for adherence to antiretroviral therapy (ART).

*The Suubi+Adherence* study recruited 702 YLHIV. Inclusion criteria included: 1) tested positive for HIV (confirmed by medical report and aware of status); 2) living within a family; 3) being 10–16 years of age (at enrolment); 4) having been prescribed ART; and 5) enrolled in ART care at one of the 39 health clinics/centers in the greater Masaka Region of Uganda -a geographical region hardest hit by HIV/AIDS (prevalence 10.6% vs. 7.2% national average) [131–133]. Health clinics were eligible if they: 1) had existing procedures tailored to adolescent adherence (including adolescent-specific clinic days and peer counselling) and 2) were accredited by the Uganda Ministry of Health as a provider of ART within the study districts.

Following enrolment and baseline assessments, 39 participating clinics were randomly assigned to one of two study groups, resulting in 19 clinics (n = 344 participants) assigned to the control condition and 20 clinics (n = 358 participants) assigned to the intervention condition. A two-arm, cluster randomized trial design was employed where all participants attending the same clinic were assigned to the same study group. All participants received medical Standard of Care (SOC), as defined by the Uganda Ministry of Health guidelines for pediatric and adolescent HIV care and treatment; and psychosocial SOC, consisting of information leaflets on adherence and support provided by lay counsellors including persons living with HIV who have been trained in ART adherence counselling, known as “expert clients”. Due to inconsistency in which SOC is often provided in the region, *Suubi+Adherence* provided youth in both study conditions with a bolstered standard of care (BSOC), which included eight information sessions on adherence to ART, adapting evidence-based, print cartoons to portray adherence topics in a relatable manner [134]. Adolescents in the intervention group received the BSOC as well as a FEE intervention consisting of a Youth Savings Account (YSA), matched at a rate of 1:1 and from which financial savings could be used for medical expenses, family microenterprise development or education related expenses (including school lunches and fees). As part of the YSA package, the intervention group also received four workshops blending FLT and life skills with topics on asset building, IGA, goal setting, and risk mitigation. Data was collected at baseline, 12, 24, 36 and 48- months post-intervention initiation. The study retention rate of 93.4% over a 6-year period (2012-2018).

### Current study

**Fig. 1:**
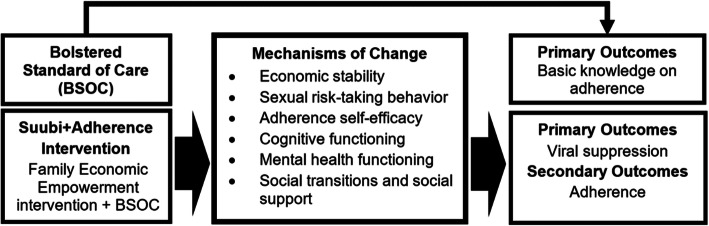
Suubi+Adherence-R2 Conceptual Framework

This study will examine the longitudinal HIV antiretroviral therapy (ART) adherence and related outcomes, as well as the potential mechanisms of protective health behaviors among youth living with HIV (YLHIV) who participated in an evidence-based family economic empowerment (FEE) intervention in rural Uganda and are now transitioning into young adulthood (see figure 1).

The research team will rely on the completed future contact forms of the *Suubi+Adherence* participants and the community trust, including with the health clinics and Masaka Diocese, for tracking and following the *Suubi+Adherence-R2* participants. The future contact information completed at the time of enrollment provides the ability to contact them, even if they have moved. Participants will be followed up within their original study conditions. For treatment group participants, we will request their bank statements each quarter to ascertain their savings. No additional matching will be done.

### Inclusion Criteria

**Fig. 2:**
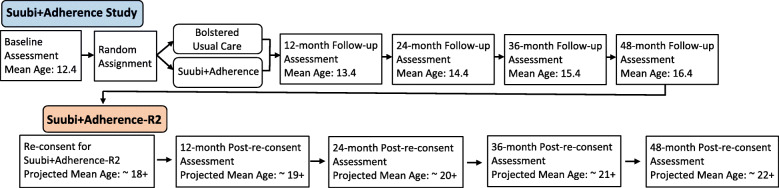
Suubi+Adherence and Suubi+Adherence-R2 Follow-up Timeline

All participants in the *Suubi+Adherence* study will be eligible for re-consenting and re-enrollment into the Suubi+*Adherence-R2* study and followed for an additional 5-year period. A recent follow-up with the 39 clinics (December 2019) by Washington University’s International Center for Child Health and Development (ICHAD) office in Uganda confirmed that with conservative estimates, we will locate at least 80% of the original sample. Following reconsenting, participants will be interviewed for 5 years, once a year. Figure 2 provides the timeline.

### Ethics and Informed Consent

The study received approval from the Institutional Review Board (IRB) at Washington University in St. Louis (IRB # 201704066) and the in-country local IRBs in Uganda: Makerere University School of Public Health Review Committee (Protocol # 210), and Uganda National Council of Science and Technology (UNCST, SS 2969). All potential amendments to the study protocol will be submitted for approval to the above-mentioned IRBs by the MPIs.

Written informed consent will be obtained from all participants. They will be assured that participation is voluntary and medical care will not be affected. Procedures for confidentiality, including handling of data, Certificates of Confidentiality, and HIPAA will be explained. Recruitment and consent procedures are all based on successful procedures from Suubi+Adherence. Since all participants will be 18 or older, we will not need caregiver consent to participate in Suubi+*Adherence-R2*.

### Sample Tracking, Retention and Attrition

The *Suubi+Adherence* study (2012-2018) had an attrition rate of 6.6%, leaving a total study sample of 656 – by study end (December 2018). These numbers reflect: a) the study being implemented in a stable region with low geographical moves; b) trust of the study team by the community members. This trust has been built and/nurtured over a 15+ year period that Dr. Ssewamala (Lead PI) and his colleagues have been engaged in the greater Masaka region and the surrounding districts; c) Washington University has a fully-fledged field office (ICHAD) in the Masaka region allowing for easy engagement between the study team and the local collaborators; and d) the study team has a well-established tracking system implemented by ICHAD resulting in very low attrition rates—as reflected in Table 1 below, detailing actual retention rates from the *Suubi+Adherence* original study.

**Table 1:** Suubi+Adherence Retention rates: (2012-2018) [48, 49, 135]

Baseline/Wave 1	Wave 2	Wave 3	Wave 4	Wave 5
*N*=702	*N*=670, retention rate=95.4% (lost to follow-up *n*=32)	*N*=663, retention rate=94.4% (lost to follow-up *n*=39)	*N*=662, retention rate=94.3% (lost to follow-up *n*=40)	*N*=656, retention rate=93.4% (lost to follow-up *n*=46)

During re-consenting, we will ask participants to update their future contact information with updated telephone number(s) (if they have one: 95% of participants reported having access to a phone in the household), and an updated list of the names, and contact information of three people who will always know how to reach them. Participants will be reminded that if we contact the people listed, we will not discuss any details about them or their study participation. Like we have done in the past, we will use these records solely to help track their location and only if we have lost contact. We have effectively used these procedures in our previous research studies, resulting in very low attrition rates. Thus, we conservatively expect to re-consent at least 80% of the 702 participants (N=562) for the Suubi+*Adherence-R2* study. We expect attrition from reconsented cohort (wave 6, in 2020) to the end of the follow-up period (wave 10 in 2025) to be no more than 20%. (See Table 1 above).

We will keep careful records of those who drop out of the study and test for attrition bias based on data we will have prior to participant drop-out. To the extent that such bias is present, we will limit generalizations accordingly, or, where possible, introduce statistical adjustments to address bias. These strategies follow the general protocol used in our earlier NIH funded studies in the study area [74, 96, 98, 102, 124–126, 136–139], including the 6-year *Suubi+Adherence* study [3] which yielded a high retention rate (93.4%) over a 6-year period, and other Suubi [140–144] and the Bridges studies (retention rate: 92.7% over 5 years) conducted by the team [125, 139, 145, 146]. Combined with the original *Suubi+Adherence, Suubi+Adherence-R2* will be one of the most complete longitudinal data sets on behavioral health in YLHIV within a FEE intervention framework. This will allow us to generate knowledge on the long-term impact of FEE interventions on YLHIV, especially during social transitions.

### Data collection

We will use self-report questionnaires with read-aloud procedures by staff to address issues of literacy. Multiple research assistants (RAs) will be hired by ICHAD to conduct assessments. To encourage truthful responding, we will remind participants that responses are confidential and re-explain to them the security system that makes it impossible to link a name to data. Each participant will complete five interviews, 12 months apart. We selected this frequency in consultation with the clinics, statisticians, and behavioral experts to balance the scientific value of capturing change through frequent interviews, with the needs of participants and clinics and with our concerns about burden, data quality, and attrition. The 12-month interval between assessments in the original *Suubi+Adherence* proved sufficiently short enough to capture critical information, but long enough to avoid response bias or problems with retention or participation.

The complete battery will be administered at all time-points and participants will be interviewed individually, which will last between 60-90 minutes. The battery takes into account: 1) sensitivity to participant’s literacy (RAs read questions aloud and help fill out measures); 2) need for trust and rapport, and 3) use of local phrases and terms. In our previous work, participants completed these interviews without incident [74, 96, 98, 102, 124–126, 136–139], often reporting positive experiences in being interviewed. If participants express distress or issues of confidentiality emerge, we have developed an emergency protocol successfully used in *Suubi+Adherence* and other ICHAD studies conducted in the region [3, 74, 96, 98, 102, 124–126, 136–139, 142–144]. All assessments will take place in ICHAD’s private research field offices in Masaka; at satellite sites: MildMay, RTY-Uganda (see Letters of Support); or at the participants’ homes (if they request it and there is sufficient privacy to ensure confidentiality. Flexibility in interview location has been critical to Suubi+Adherence and other ICHAD studies success to date, with approximately 40% conducted in research offices in Masaka, and 50% in satellite sites. All measures (Table 2) have been pre-tested in the *Suubi+Adherence* study and other ICHAD studies [5, 9, 32], and made culturally appropriate to the Ugandan context. The added cognitive tests have also been previously validated in Uganda with YLHIV [19, 20].

**Table 2:** Suubi+Adherence-R2 Study Measures

Variable	Measurement	Reliability	Timepoint
Demographics (Respondent: Adolescent Participant)			
Age; sex (assigned at birth), orphan status (single vs. double); socioeconomic status; family composition/structure; caregiver educational level	Socio-demographic questionnaire	n/a	^a^T1-RC, 12, 24, 36, 48
Moderators (Respondent: Adolescent Participant)			
Rural/urban/semi-urban; exposure to outside HIV/STI-related programs; economic/household income; asset accumulation			^a^T1-RC, 12, 24, 36, 48
Potential Mechanisms of Change (Respondent: Adolescent Participant)			
Self-efficacy	Adapted Tennessee Self-Concept Scale [147] (TSC-2) (*pretested in Suubi Studies*)	0.81	^a^T1-RC, 12, 24, 36, 48
Education plans/aspirations	Adapted Monitoring the Future Survey [148]	n/a	^a^T1-RC, 12, 24, 36, 48
Motivation to participate	Questions tested in previous studies [103, 124, 149, 150]	n/a	^a^T1-RC, 12, 24, 36, 48
Family Support	Social Support Behaviors Scale (SS-B) [151]Family Cohesion Scale [152], Krauss Interview [153]Parent Child Relationship Inventory (PCRI) [154]	0.77 0.69 0.91	^a^T1-RC, 12, 24, 36, 48
Family Stability	Socio-demographic questionnaire	n/a	^a^T1-RC, 12, 24, 36, 48
Hopelessness	Beck Hopelessness Scale [152]	0.79	^a^T1-RC, 12, 24, 36, 48
Self-esteem	Rosenberg Self-Esteem Scale [155]	0.77-0.88	^a^T1-RC, 12, 24, 36, 48
Condom Negotiation Self-Efficacy	Condom negotiation self-efficacy scale [156]	0.80	^a^T1-RC, 12, 24, 36, 48
Sexual Communication Skills	Sexual Communication Scale [157]	0.80	^a^T1-RC, 12, 24, 36, 48
Transitions (education, employment, residential independence, prosocial transition, relationship/marital status, pregnancy/parenthood	Demographics, MTF, Work history interview, Relationship assessment scale, Adolescent Sexual Behavior Assessment (ASBA), additional prosocial activities		^a^T1-RC, 12, 24, 36, 48
Social Support	Multidimensional scale of perceived social support MSPSS [158]	0.84	^a^T1-RC, 12, 24, 36, 48
Access to services	RBA Services [159], Treatment Services Review (TSR) teen and adult versions [160–162]	.66-.83	^a^T1-RC, 12, 24, 36, 48
Cost of staff time, supplies, overhead for IDAs	Project records; Admin. Review	n/a	ongoing
Substance misuse	Questions adapted from PhenX Toolkit [163], Alcohol, Smoking, and Substance Involvement Screening Test [164], DISC-IV modules, CIDI modules	n/a; 0.58-0.90	^a^T1-RC, 12, 24, 36, 48
HIV stigma/disclosure	CASAH Social Disclosure Interview [165], Social impact scale [166]		^a^T1-RC, 12, 24, 36, 48
Cognitive function	Peabody Picture Vocabulary Test (PPVT) [167], Symbol Digit Modalities Test (SDMT) [168], Color Trails Test [169], WHO_UCLA Auditory Verbal Learning Test [170], Grooved Pegboard Test [171]	0.61-0.88, 0.76, 0.64-0.79, 0.66, 0.85-0.91	^a^T1-RC, 12, 24, 36, 48
**Primary Outcomes (Respondent: Adolescent Participant)**			
Viral load/Virologic Suppression*	Biomedical data: Viral load*	n/a	^a^T1-RC, 12, 24, 36, 48
Secondary Outcomes (Respondent: Adolescent Participant)			
Adherence	Self-Report Questionnaire, Pill Counts, adherence self-efficacy [172]		^a^T1-RC, 12, 24, 36, 48
Financial/economic stability (savings and asset accumulation)	Bank statements, MIS IDA, and American Dream Policy Demonstration [173]	n/a	^a^T1-RC, 12, 24, 36, 48
Sexual risk taking behavior:	Time-line Follow Back (TFLB) [174, 175] Adapted Youth Aids Prevention Project (used in Suubi & CHAMP) [176]	n/a	^a^T1-RC, 12, 24, 36, 48
Mental Health Functioning	Center for Epidemiological Studies-Depression Scale (CES-D) [177]	0.65	^a^T1-RC, 12, 24, 36, 48
STIs	Biomedical data: Gonorrhea, Chlamydia, Trichomonas	n/a	^a^T1-RC, 12, 24, 36, 48

^a^T1-RC – Time 1 at reconsenting.

#### Biological assay

To measure Viral load (VL) suppression, we will use the same procedure from *Suubi+Adherence* for Suubi+Adherence-R2: Viral load testing will be done by blood draws at 12-month intervals post-reconsenting. In accordance with the Abbott platform, VL will be dichotomized between undetectable (< 40 copies/ml) and detectable (≥ 40 copies/ml) levels.

In addition to VL, to assess sexual risk-taking behaviors, a secondary outcome, we will test for Gonorrhea, Trichomonas, and Chlamydia. In conducting these tests, collection, counseling, notification, referral for treatment, follow up and monitoring procedures for these biological test—all used in current studies by Ssewamala and colleagues – will be completed at Waves 6 – 10. As recommended by the CDC, we will use the more sensitive NAATs for Chlamydia and Gonorrhea testing. Trichomoniasis will be tested using culture which is considered a current criterion standard. Samples for culture will be obtained using vaginal swabs.

#### Adherence

ART adherence will be assessed by three items that have been used widely in resource limited settings with low-literacy populations addressing: 1) how often they take all of their HIV medications as prescribed, 2) their ability to take all of their medications as prescribed, and 3) how often they missed taking their medications within the past 4-weeks and 6-months [178]. This will be augmented with unannounced pill counts to be conducted at least once every 3 months – as used in *Suubi+Adherence*.

Unannounced pill counts conducted in patients’ homes have demonstrated validity for monitoring medication adherence [3]. Specifically, using the *Suubi+Adherence* protocol for unannounced pill counts [3], the Research Assistant (RA) will follow the following steps [3]: 1) upon visiting the home, ask the participant to bring out all medications in the home, including closed bottles, pillboxes, and doses kept in pockets, purses, backpacks, etc.; 2) ascertain each participant’s regimen before actual counting; 3) in the presence of the participant, thoroughly clean their hands using soap and water, dry their hands using a clean dry towel; 4) using alcohol prep wipes, clean a pharmacy pill tray—that the RA will have– to be used for pill counting; 5) sort the medications into clusters using a spatula; 6) record prescription numbers on each medication, refill date, and dispensed quantity; 7) ask the participant if they had lost or received any pills since their previous count and whether they had taken any of the drug that day; 8) in the presence of the participant, count pills using the study-provided pharmacist tray; and 9) repeat the procedure to double count. Participants will provide times of days they are most likely to be available for pill counts. We will visit participants at an undisclosed time and day to count their pills once every 3 months throughout the study period. Adherence is calculated as the difference between the current and previous pill count divided by the prescribed number of doses for the same period. We have used the same procedures in our *Suubi+Adherence* study. Unannounced pill counts by RAs have been validated in multiple studies with strong association with VL, and has had limited, if any, impact on adherence behaviors [179].

#### Semi-structured qualitative interviews

Two sets of qualitative interviews will be conducted. The first set will be at Wave 6 (Immediately following re-consenting) to explore: 1) participants’ previous experiences with the FEE intervention and its specific components for the treatment group, and their experiences with BSOC for the control group; 2) key multi-level factors (individual, economic, family, contextual, and programmatic) that may have impacted their participation; and 3) key multi-level factors that may have affected their decision-making and behaviors around ART adherence as well as overall mental health since the completion of the *Suubi+Adherence study*. The second set of interviews will be at each follow-up time point (Waves 7-10) to unpack: 1) the longer-term impact, including key multi-level factors affecting participants’ adherence and savings decisions, and mental health as they transition to young adulthood; 2) experiences with social transitions and their potential effect on decision-making around adherence and on mental health; and 3) participants’ perceptions of economic costs and rewards of adherence. A stratified purposeful sampling strategy [180] will be used to randomly select participants from each quartile. Participants in the highest and lowest quartiles on three key outcomes (sexual risk-taking, savings, and mental health) will be invited for interviews (n=80). This sample size will achieve theoretical saturation [181, 182] and will represent ~15% of the participants in each study condition (n=40 for treatment arm; n=40 for BSOC arm). This sampling method will ensure that participants with varying experiences are represented and allow us to identify common patterns and variations in their experiences. Interviews will be conducted in English or Luganda based on participants’ preference. Questions will be translated (English to Luganda) and back-translated by two team members. Questions will be reviewed by two native speakers to ensure they sound natural and conversational. Each interview will last about 60 minutes and will be audio-taped.

### Data Analysis Plan

#### Data quality assurance, initial analyses, and missing data

We will continue to use MIS IDA Q [75] to check for data-entry errors and missing values. Frequency tables for all variables and measures of central tendency and variability for continuous variables will characterize the sample overall and by randomization group. We will address incomplete data with direct maximum likelihood (ML) and multiple imputation (MI) [183] because they make the relatively mild assumption that incomplete data arise from a conditionally missing-at-random (MAR) mechanism [184]. Auxiliary variables will be included to help meet the MAR assumption [185, 186] and sensitivity analyses will be conducted with weighted MI [187] to assess the robustness of the MAR assumption [188]. SAS [189] and M*plus* [190] will be used to perform the analyses.

#### Time points used in inferential analyses

To maximize rigor, to assure alignment of cognitive functioning data (only measured in the *Suubi+Adherence-R2 study*) and our remaining outcomes, and to avoid reuse of *Suubi+Adherence* outcomes (already examined for shorter-term intervention efficacy) that would bias tests of longer-term intervention efficacy, all primary inferential analyses and most of the secondary inferential analyses listed will use only Suubi+*Adherence-R2* data. The exception is longitudinal developmental trajectory analyses to explore associations between changes in time-varying covariates and mechanisms of change with outcomes measured across both *Suubi+Adherence* and *Suubi+Adherence-R2*.

#### Primary Analyses for Aim 1

We hypothesize that participants in the *Suubi+Adherence* intervention group will exhibit a higher odds of HIV viral suppression relative to participants in the bolstered standard of care (BSOC) control condition (H1a) during Suubi+*Adherence-R2*. To test H1a, we will fit a three-level generalized linear mixed model (GLMM) with fixed effects for study arm, time, and their interaction. We will use random intercepts for Clinic ID to account for clustering of persons within clinics and include random intercepts, random slopes, and their covariance for person ID to account for clustering of repeated measurements within persons. Reflecting the binary HIV viral suppression outcome, a binomial distribution and logit link will be used. To test H1a we will perform a time-averaged comparison of repeatedly measured observations across study arms to examine intervention effects over the duration of the study period. Alpha will be set at .05 for this planned comparison.

#### Secondary Exploratory Analyses for Aim 1

We will employ the same three-level GLMM strategy as described above to evaluate the exploratory hypotheses that assess whether the odds of ART adherence and engagement in HIV care are higher in the *Suubi+Adherence* intervention group to the BSOC control group. We will investigate in secondary exploratory analyses whether *Suubi+Adherence* intervention group participants exhibit during Suubi+*Adherence-R2*: H1(b) a higher odds of adhering to the prescribed medication regimen at > 90%; and H1(c) increased odds of keeping all medical appointments in the last 12 months. The same three-level GLMM approach as described above for the primary analysis will be used. Alpha (α) will be set to .05 for each of these exploratory hypotheses. To maximize rigor, quasi-likelihood methods will not be used [191]. Instead, maximum likelihood estimation via adaptive Gaussian quadrature with 15 integration points will be used to ensure stable solutions.

#### Primary Analyses for Aim 2

We anticipate that in the long run and during the social transition period for participants, YLHIV in the intervention group will report superior mean levels of protective health behaviors, improved financial outcomes related to successful uptake of the microfinance-based principles FEE, and lower negative health risk behaviors, as implied by Specific Aim 2. Relative to participants in the BSOC control condition, intervention group participants should, over time/during the transition period: H2(a) exhibit lowered mean levels of sexual risk behavior; H2(b) report increased mean levels of adherence self-efficacy; H2(c) increased mean economic stability; H2(d) increased mean levels of cognitive functioning, and H2(e) increased mean levels of mental health functioning, H2(f) increased mean levels of social support and H2(g) prosocial transition functioning. To test H2(a)-H2(g) we will fit linear mixed models (LMMs) using the same fixed effects (study arm, time, and study arm-by-time) and random effects for the clinic (random intercepts) and person levels (random intercepts, random slopes, and their covariance) as in the proposed H1 analyses described. To maintain a nominal Type 1 error rate of 5% across tests of H2(a)-H2(g), α will be set at .05/7=.007 for each planned time-averaged comparison. To maximize rigor, the assumptions of normality and constant variance of residuals for these continuous outcomes in LMMs will be evaluated by examining histograms of the residuals and scatter plots of predicted values-by-Cholesky-scaled residuals, respectively. Transformations of outcomes will be utilized as needed to improve data conformance with model assumptions. Inferences for models whose residual statistics still do not fully meet assumptions following transformations will be generated via robust heteroskedastic-consistent estimators [192]. All analyses will include outlier and influential case screening via computation of Cook’s D, DFBetas, and likelihood displacement statistics. If outliers are found, results will be reported with and without outliers included [193, 194].

#### Secondary exploratory analyses for Aim 2

The same GLMM approach described above for Aim 1’s primary analysis will be used to test whether the odds of STI acquisition during young adulthood is lower across time for intervention participants than control participants.

##### Exploring mediation and moderation

To explore pathways by which the *Suubi+Adherence* intervention effects change in outcomes, secondary analyses will investigate whether the potential mechanisms of change listed in Table 1 mediate the relationships between intervention group assignment and sexual risk, HIV viral suppression, STI, mental health outcomes, and neurological functioning, and whether geographic location, exposure to other HIV/STI prevention programs, household income and asset accumulation moderate those associations. Mediation and moderation will be assessed using the causal inference-based approach of Valeri and VanderWeele, which yields optimal estimates of indirect effects in the presence of non-continuous (e.g. binary) outcomes and moderator-mediator interactions [195]. M*plus* will be used to fit mediation models because it can adjust standard errors for clustering of participants within clinics [196].

##### Exploring trajectories of change over the full developmental period captured by the Suubi+Adherence project

Moderator, mediator, and sexual behavior and mental health outcome data will be available at 10 time points from the start of the original *Suubi+Adherence* through the end of the *Suubi+Adherence-R2* study. We will initially explore trajectories of these variables by generating spaghetti plots of participants’ individual trajectories with study arm averages overlaid to visualize group average (i.e., mean) levels of change and inter-participant variability. Next, we will fit mixed effects growth models to learn whether change over time can be quantified using low-dimensional parametric functions (e.g., linear, quadratic) of explanatory variables. Finally, we will employ the highly flexible time-varying effect model (TVEM) to go beyond simple parametric functions to elucidate the form of variables’ changes over time. TVEM allows the effects of covariates on outcome trends to vary over time non-parametrically [197]. TVEMs will be fitted using the %weightedTVEM SAS macro from the Penn State Methodology Center, a leader in developing longitudinal analysis methods and software. The %weightedTVEM [198] macro includes support for clustered data, enabling us to account for clustering of participants within clinics. Moderation by intervention group will be featured in all trajectory analyses to estimate separate slopes or curves for control and *Suubi+Adherence* treatment groups.

##### Sex as a biological variable

In line with recent NIH guidelines on exploring sex differences in effects, all previously described inferential analyses will be repeated with models extended to include sex assigned at birth as a moderator to examine whether effects vary by participant sex.

#### Statistical Power Analysis for Aims 1 and 2

We used NCSS PASS [199] to compute minimum detectable effect size estimates for the primary hypotheses H1(a) and H2(a)-H2(g) proposed to fulfil Specific Aims 1 and 2, respectively. For all power analyses we assume power=.80 and 5 repeated assessments from N=450 participants conservatively assuming 20% attrition. For H1(a), based on our current *Suubi+Adherence* project’s data we further assumed a control proportion of 65% virally suppressed, α=.05, and an intraclass correlation (ICC) of .032 at the clinic level and .819 at the person level. Under these assumptions, the minimum detectable raw proportion difference is 13%, which corresponds to an odds ratio (OR) of 1.91 and a standardized effect estimate *h*=.29, which is between a small (*h*=.20) and medium (*h*=.50) effect size [200] The minimum detectable OR of 1.91 is less than ORs obtained in the *Suubi+Adherence* study [6] ensuring that we will have sufficient power to detect similar or slightly smaller effects in the project period. For H2(a)-H2(g), we computed the minimum detectable standardized mean difference *d* using the same inputs as listed above, α=.007, and a range of ICCs from .003 to .542 for change mechanisms based on data from the *Suubi+Adherence* study. Under these conditions *d* ranged from .10 to .13, which are small standardized effect sizes [201]. Taken collectively, our study will have sufficient power to detect small to small-medium effects across a wide variety of conditions.

#### Qualitative data analysis for Aim 3

All interviews will be transcribed (and translated to English where necessary). Transcripts will be compared with digital records to ensure transcription accuracy. All transcripts will be uploaded to QSR NVivo12 analytic software for analysis. Interview transcripts will be reviewed by the research team to develop a broad understanding of content as it relates to the project’s aims and to identify topics of discussion. During this step, as well as during subsequent steps, analytic memos will be written to further develop categories, themes, and subthemes, and to integrate the ideas that emerge from the data [202–204]. Using analytic induction techniques [205], transcripts will be read multiple times for initial coding by the research team. For initial coding, randomly selected 10 interview transcripts will be read multiple times and independently coded by the project investigators using a priori (i.e., from the interview guide) or emergent themes (also known as open coding) [202, 206]. Broader themes/categories will be broken down into smaller, more specific units until no further subcategory is necessary. For example, potential themes and subthemes for barriers and facilitators to adherence may include: at the individual-level (e.g., motivation, readiness to change, savings, household income), family-level (e.g., support); and macro-level (e.g., cultural norms, stigma). These findings will also further contextualize the mediators and moderators and may identify additional variables to be considered as part of mechanisms of change.

The codes and definition boundaries of specific codes (inclusion and exclusion criteria for assigning a specific code) [207] will be discussed during team meetings to create the final list of codes (known as codebook) in NVivo. Each text will be independently coded by two investigators using the codebook. Inter-coder reliability will be established. A level of agreement from 66 to 97% depending on level of coding (general, intermediate, specific), indicates good reliability in qualitative research [203]. Disagreements will be resolved through discussion during team meetings. The secondary analysis will focus on comparing and contrasting themes and categories within and across groups to identify similarities and differences). To further ensure rigor, study results will be presented to study participants, enabling them to provide comments and suggest modifications or additional avenues of investigation when possible (member checking) [204, 208]. An audit trail [204, 208] of data as well as memos and minutes of team meetings will be kept throughout the study.

#### Cost-effectiveness (CE) analysis for Aim 4

CE analyses measure the cost of an intervention relative to other means of achieving the same desirable outcome. Following standard practice, we will measure costs on a per person basis. The costs of the intervention include all costs for running program activities. Research costs will be excluded. Data on the costs of other program elements will be drawn from project administrative records. Outcome analyses described above will be used to assess the extent to which *the intervention*, in the long-run, affected particular outcomes. The per-person costs of *the intervention* will then be divided by the relevant effect sizes to estimate CE ratios. For example, suppose that, over the 10-year period, *the intervention* costs per child were respectively $500 and $750 and, on average, increased years of schooling completed by one year and two years. The CE ratios described above (section C.2.3) will, like the effect sizes from which they derive, be point estimates. Confidence intervals based solely on effect size confidence intervals ignore the cost portion of CE. But cost estimates are also only point estimates on a per person basis. We will calculate confidence intervals using two methods [209, 210]: Monte Carlo [211] and bootstrap [211]. Finally, for some outcomes, such as increases in education and health, we will be able to compare the CE of *the intervention* to other interventions in developing country settings [212–218].

#### Data integration

The qualitative and quantitative data analyses will be done separately. Findings will be integrated at the interpretation and discussion stages. Conclusions and inferences will be synthesized for a more contextualized and thorough understanding of intervention impact. Data integration will serve two purposes: 1) *Complementarity* [219, 220]; and 2) *Expansion* [219, 220]. Qualitative and quantitative findings will be connected, where the former will provide explanations and context for findings produced by the latter (complementarity). The qualitative data will potentially provide further explanation and context for the observable study results.

### Monitoring and Responding to Adverse Events

We will identify, manage, and document events and psychological distress reactions that actually occur during the study period. These events may be identified by project staff or reported by participants. All study personnel, including data collectors and facilitators will receive extensive training on how to identify verbal and non-verbal signs that may indicate psychological distress and adverse events. They will also be trained on how to support distressed participants and to offer referrals to local clinics/ hospitals if necessary. The in-country teams at RTY, Mildmay and ICHAD are knowledgeable of resources available to participants in the study region. If the need arises, RAs will make appropriate referrals for basic and enhanced services.

Reporting of adverse events will occur according to a project protocol. For this study, safety and monitoring will be overseen by three MPIs (Drs. Ssewamala, Sensoy Bahar, Nabunya), based in the United States. This group is expected to have weekly telephone conference calls (using skype or zoom). In the case of an adverse event, staff will inform the MPIs (Drs. Ssewamala, Sensoy Bahar, Nabunya) immediately. Any presence of a possible unanticipated adverse event will be immediately reported and brought to the attention of the Washington University Institutional Review Board (along with the Ethics Committee at UVRI and Uganda National Council of Science and Technology). The IRBs will determine whether it is appropriate to stop the study protocol temporarily or will provide suggestions and/or modifications to the study procedures. Possible modifications may include adding new risks to the consent form and re-consenting all study participants.

Preliminary outcomes data will be examined quarterly by the MPIs and the Co-Is. If preliminary outcome data indicates harmful impact of the program to the study participants, Washington University IRB committee, as well as the Ethics Committee at UVRI and Uganda National Council of Science and Technology IRB will be notified and it is possible that the study will be discontinued immediately. However, we do not anticipate any negative effects of participating at this time.

### Data Management and Integrity to Protect Confidentiality

To protect the integrity of the participants' data, the following procedures will be followed. First, the data collected from the study participants will be used only for the purpose of research. All data will be kept confidential. We will not share any information or answers we get from the participants with their families, friends, health clinic workers. Since the study is a longitudinal one, participants will keep the originally assigned study IDs from the *Suubi+Adherence study*. We maintain lists of participants with links between identifying information and code numbers. Only the MPIs and the in-country Project Coordinator will have access to these lists, which are kept in locked files. Other study personnel have access on an as needed basis to individual participants' names and code numbers in order to adequately perform their duties, for example, interviewers must label the questionnaires with the correct code number of the participant whom they are interviewing. All personnel must complete certain levels of training before they are granted access to this identifying information. They must complete the Human Subjects Training sponsored by the National Institute of Mental Health, which complies with federal guidelines delineated in 45 CFR Part 46. Personnel also sign confidentiality statements that specify that if the participants' confidentiality is breached unintentional that personnel will follow the procedures for reporting this breach to the MPIs. The confidentiality statements also state that unintentional or deliberate violations of participants' confidentiality may result in demotion or termination depending upon the severity of the event. The project personnel also participate in training with the MPIs and the in-country Co-I (Dr. Mukasa) regarding data safety, confidentiality of participants, limits of confidentiality, and proper administration of the study protocol. All hard copies of data will be stored in locked cabinets to which only the MPIs and the in-country project coordinator will have access. After completion of an interview with a study participant, data with code numbers is placed in a separate locked file cabinet while waiting for entry. Once data is entered into computer files and password protected, only the MPIs, the in-country Project Coordinator, and Data Entry Assistant have access to these files.

All requests, current and future, to use the data are reviewed by the MPIs. Any data files that are provided to other individuals are stripped of identifiers and contain only code numbers so that data across multiple assessment waves can be matched. Within the informed consent/assent, participants are notified of the above procedures.

The MPIs (Ssewamala, Sensoy Bahar, Nabunya) and Project Coordinator in Uganda are social workers by training. They will all be available to talk to the participants who may experience discomfort with the questions on the interviews, should they cause concern. Dr. Mukasa and the Mildmay team are medically trained and will be able to talk to participants who may experience discomfort during blood draw. In case further counseling is needed, the project staff will make the referrals.

## Discussion

To our knowledge, there are no known studies that examine the longitudinal adherence and behavioral health outcomes, and transition milestones for YLHIV in SSA in the context of economic empowerment that may be a protective factor for this group. The scientific premise for this study derives from previous research that signals that YLHIV face more challenges related to treatment adherence as well as critical developmental, psychosocial, neurological, and economic problems than the general population [221–226]. Moreover, given the stigma YLHIV experience, they are faced with limited social support and opportunities for education and employment, which elevates their vulnerability to poverty and other negative life outcomes, including poor adherence to ART regimens, virologic failure [15, 17, 221, 222, 227–230], and cognitive deficits [63, 231, 232] Thus, in *Suubi+Adherence-R2* we will build the largest known longitudinal health and wellbeing dataset (over a 10-year period) from a cohort of YLHIV who participated in a FEE intervention in a low-resource setting and are transitioning into young adulthood.

The study innovates in several ways. The study extends the evaluation of a “home-grown” intervention from a focus on short-term impacts to longer-term outcomes as adolescents transition through young adulthood—hence ascertaining the extent to which the short-term effects from FEE are sustained in the long run. To our knowledge, *Suubi+Adherence-R2* would be the first of its kind to measure the long-term efficacy of a FEE intervention for YLHIV. As such, the study will enable us to learn the extent to which participation in a FEE intervention may potentially change the would-be poor trajectory for YLHIV in low-resource communities. Suubi+Adherence-R2 will be one of few studies of YLHIV in SSA to use multiple measures of adherence. Adherence remains a primary barrier to actualizing the full potential of ART, and no assessment method has proven to be the “gold standard” or economically feasible for wide use, particularly in low resource settings. Suubi+Adherence will use virologic suppression as the primary outcome; and for secondary outcomes augment self-reports which are subject to memory and social desirability bias with use of unannounced pill counts. This will allow us to compare data from multiple measures to more accurately assess true adherence, extending work from adult HIV studies [50, 51]. Finally, Suubi+Adherence-R2 will identify significant pathways to both successful and problematic transitions and will determine strategic intervention points for YLHIV in SSA, one of the world’s poorest regions with the highest HIV prevalence rates. These results may be relevant for others from impoverished backgrounds coping with chronic health conditions for which the literature on this developmental transition is limited. New to this study will be the inclusion of cognitive functioning measures, which have previously been shown to impact ART adherence in other settings and which may be influenced positively by previous FEE intervention exposure.

The research team will facilitate learning across stakeholders and maximize use of the evidence generated through dissemination meetings. If findings warrant, this study will leverage the current Ugandan government policy guidelines regarding youth empowerment contained in the Vision 2040 framework (launched by the government on April 18th, 2013) to maximize dissemination of study findings. The study will also leverage and offer learning to the government in regards to the ART Adherence and HIV treatment Policy guidelines for adolescents and young adults.

Economically vulnerable YLHIV are less likely to adhere to ART and other health-related regimens [42–51]. As a consequence, YLHIV are not only at risk for health problems and mental health comorbidities, but are also at risk for cognitive deficits, including in areas of memory and executive functioning [231, 232]. This study addresses new scientific questions regarding long-term ART adherence; HIV care engagement [233]; protective health behaviors; and the potential of FEE to mitigate the development of HIV-associated neurocognitive disorders in YLHIV [105–107]. The findings from this study may inform efforts to improve HIV care among Uganda’s YLHIV, with potential replicability in other low-resource countries. Moreover, the proposed study is within the HIV/AIDS research high priority areas.

## Data Availability

Not available
